# Systems Biology Understanding of the Effects of Lithium on Affective and Neurodegenerative Disorders

**DOI:** 10.3389/fnins.2018.00933

**Published:** 2018-12-13

**Authors:** Weihao Ge, Eric Jakobsson

**Affiliations:** ^1^National Center for Supercomputing Applications, Urbana-Champaign, Urbana, IL, United States; ^2^Center for Biophysics and Computational Biology, Urbana-Champaign, Urbana, IL, United States; ^3^Department of Molecular and Integrative Physiology University of Illinois at Urbana-Champaign, Urbana, IL, United States

**Keywords:** lithium, systems biology, affective disorders, neurodegenerative disorders, biochemical pathways, biochemical networks

## Abstract

Lithium has many widely varying biochemical and phenomenological effects, suggesting that a systems biology approach is required to understand its action. Multiple lines of evidence point to lithium intake and consequent blood levels as important determinants of incidence of neurodegenerative disease, showing that understanding lithium action is of high importance. In this paper we undertake first steps toward a systems approach by analyzing mutual enrichment between the interactomes of lithium-sensitive enzymes and the pathways associated with affective and neurodegenerative disorders. This work integrates information from two important databases, STRING and KEGG pathways. We find that for the majority of neurodegenerative disorders the mutual enrichment is many times greater than chance, reinforcing previous lines of evidence that lithium is an important influence on incidence of neurodegeneration. Our work suggests rational prioritization for which disorders are likely to be most sensitive to lithium and identifies genes that are likely to be useful targets for therapy adjunct to lithium.

## Introduction

Lithium is typically a first line therapy for bipolar disorder, including associated depression as well as mania (Post, 2016). A comprehensive review of the literature confirms that lithium is also effective against unipolar depression with unique anti-suicidal effectiveness, and may also be useful against cancer and neurodegenerative disease (Jakobsson et al., 2017).

Significant insights have been gained into the biochemical bases of lithium's action. Much of lithium's biochemical action may be summarized by noting that it inhibits enzymes that have magnesium as a co-factor (Jakobsson et al., 2017). One such enzyme, the lithium-sensitive enzyme glycogen synthase kinase 3-beta (GSK3B) (Freland and Beaulieu, 2012) inhibits signaling induced by Brain-Derived Neurotrophic Factor (BDNF) (Mai et al., 2002). Thus, lithium would be expected to enhance activity of BDNF. BDNF may be a key bridge between affective and neurodegenerative disorders, since levels of this enzyme have been implicated in depression (Karege et al., 2002), bipolar disorder(Cunha et al., 2006; Post, 2007), and dementia (Weinstein et al., 2014). Indeed, in animal experiments, lithium was shown to induce brain-derived BDNF (Hashimoto et al., 2002). In addition, BDNF has been shown to play an important role in survival of adult and developing central neurons both in culture and *in vivo* (Ghosh et al., 1994; Jones et al., 1994; Acheson et al., 1995; Conover et al., 1995; Berton et al., 2006). The role of lithium in increasing activity of BDNF plus the role of BDNF in survival of neurons support the hypothesis that lithium might have a role to play in the treatment of neurodegenerative disease (Chuang, 2004).

Other reported research results have supported the potential of lithium for treatment of neurodegenerative disease (Forlenza et al., 2014). However, relevant clinical trials remain to be done. In the absence of clinical trial results, insights may be obtained from comparative studies on bipolar patients who have received long-term lithium treatment, and those who have not. In one such study, in an otherwise well-matched cohort of elderly (~70 years old), 5% of those on long-term lithium therapy (continuous for the previous 5 years) were diagnosed with Alzheimer's disease (AD), while 33% of those not receiving consistent lithium therapy were diagnosed with AD (Nunes et al., 2007).

Epidemiological studies on the general population are suggestive. A recent nationwide study in Denmark showed that lithium level in the drinking water was significantly correlated with incidence of dementia, with higher lithium levels showing lower levels of dementia (Kessing et al., 2017). A more recent epidemiological study in Texas showed a similar specific effect for AD (Fajardo et al., 2018). An important feature of the epidemiological studies is that they involve levels of lithium ingestion that are many times smaller than those used for bipolar therapy, and are therefore almost certainly without significant side effects.

One neurodegenerative disorder, frontotemporal dementia (FTLD), initially presents with behavioral symptoms resembling mania (Woolley et al., 2011), posing a challenge for diagnosis. A definitive diagnosis in the early stage of the disease requires neuroimaging (McMillan et al., 2014). The consensus is that FTLD is invariably fatal, with a more rapid progression than AD (Roberson et al., 2005). However, there may be one documented apparent exception to the incurability of FTLD, in a case history presented by Monji et al. (2014). In this study a middle-aged man presented manic symptoms that had no apparent origin in early life. Because imaging revealed abnormalities typical of FTLD, a diagnosis of FTLD was made. However, because the psychiatric symptoms had a pattern like bipolar disease, lithium therapy was begun. In a little under 2 years the psychiatric symptoms had been completely mitigated and new brain images appeared normal. The authors concluded that the initial diagnosis of FTLD was in error. However, the data presented in the paper were also consistent with the hypothesis that the FTLD diagnosis was correct and that the lithium therapy reversed the course of the disease. Dr. Monji, first author on the study, confirmed in an email to us that this hypothesis was consistent with their data.

A case history suggests efficacy of lithium for alleviating agitation and psychosis in both FTLD and AD (Devanand et al., 2017). The efficacy of lithium for FTLD patients is to be tested in a recently announced clinical trial^1^, although only with respect to relief of the behavioral symptoms cited in the above reference over the course of a 12-week trial. The limited scope of this study is a continuation of a line of thought that considers affective and neurodegenerative aspects of FTLD as relatively separate (Huey et al., 2006), a line of thought that we question because of the evidence discussed above.

Dysfunction of autophagy is strongly implicated in neurodegenerative disease (Hara et al., 2006; Komatsu et al., 2006; Nixon, 2013; Menzies et al., 2017). Lithium has been shown to induce autophagy, due to its inhibition of inositol monophosphatase (Sarkar et al., 2005). This is the basis of a pathway for autophagy enhancement, independent of the well-studied effects of mTOR on autophagy (Kim and Guan, 2015). This additional pathway for autophagy enhancement has led to the suggestion of a combined lithium-rapamycin treatment for Huntington's Disease, with lithium inhibiting inositol monophosphatase and rapamycin inhibiting mTOR (Sarkar et al., 2007).

The full range of lithium effects on autophagy is complicated (Motoi et al., 2014), as might be expected because of lithium's lack of specificity.

Because lithium affects many different biological molecules and processes (Jakobsson et al., 2017), it is essential to utilize the tools of systems biology (Kitano, 2002) if a comprehensive understanding of lithium action and its prospects for therapy are to be obtained. Important concepts for organizing biological information in a systems context are pathways and networks. A very useful tool for obtaining data about known pathways is the KEGG database (Kanehisa et al., 2016). An equally useful and complementary tool is the STRING database of interacting proteins (Szklarczyk et al., 2016).

In the present paper we investigate further the possible linkages among (1) lithium, (2) affective disorders, and (3) neurodegenerative disorders by analyzing the mutual enrichment between STRING-derived interactomes of lithium-sensitive enzymes, and the KEGG pathways associated with affective and neurodegenerative disorders.

### Methods

Analysis was performed on the interactomes of lithium-sensitive genes, as identified by prior literature search (Jakobsson et al., 2017). This search suggested BDNF, BPNT1, DISC1, DIXDC1, FBP1, FBP2, GSK3A, GSK3B, inositol monophosphatases (IMPA1, IMPA2, and IMPAD1), INPP1, and PGM1 as key to understanding the broad biological actions of lithium. The interactomes of these genes were extracted from the STRING database (https://string-db.org). The search within STRING is adjustable with respect to two parameters; (1) The confidence level associated with each interaction and (2) What level of interaction each of the returns has with the lithium sensitive gene—first order direct interaction or second order interaction through one intermediary. For each key gene, we adjust confidence level and order of neighbors (nearest only or next nearest included), so that each set contains a few 100 genes. This size is large enough for statistically reliable enrichment analysis. Table 1 shows the minimum confidence level and the maximum order of interaction (direct, removed by one, etc.) for each set. Very similar sets were merged; in particular FBP1 and FBP2 were merged into one set, and the inositol monophosphatases were merged into one set. On the other hand, GSK3A and GSK3B showed sufficient differences to be considered separately. Overall, we consider 10 distinct lithium-sensitive entities.

**Table 1:** Interactome parameters and sizes for lithium-sensitive genes.

**Gene**	**Confidence level**	**Order of neighbor**	**Interactome size**
BDNF	0.4	1	335
BPNT1	0.6	2	388
DISC1	0.8	2	113
DIXDC1	0.6	2	378
FBP1	0.9	2	175
GSK3A	0.4	1	307
GSK3B	0.4	1	225
IMPAD	0.9	2	504
INPP1	0.7	2	228
PGM1	0.4	1	176

### Disease Association

We used the R-package KEGGgraph (Zhang and Wiemann, 2009; Zhang, 2017) to identify the genes associated with the pathways of interest. For one condition, bipolar disorder, there was no annotated pathway in the KEGG database. In lieu of an annotated pathway, we used the list of bipolar-related genes compiled by Nurnberger et al. (2014)

### Empirical *p*-Value Calculation

The fundamental question we address is whether there is significant overlap or mutual enrichment between the interactomes of lithium-sensitive genes and the pathways or gene sets implicated in affective and/or neurodegenerative disorders.

For each of the 10 lithium sets, an ensemble of 1,000 null sets are generated by random selection from the human genome. Each null set is the same size as the corresponding lithium set. Then we used the R-package STRINGdb (Franceschini et al., 2013) to perform KEGG pathway enrichment analysis. This operation is a particular example of the powerful technique of gene-annotation enrichment analysis (Huang et al., 2008). In gene-annotation enrichment analysis a test list of genes (often derived from gene expression experiments) is compared to an organized database of gene annotations, often referred to as a gene ontology (Thomas, 2017), an array of gene lists corresponding to different biological functions, molecular functions, or locations in the cell. The output of the gene-annotation enrichment analysis is expressed as the likelihood that the list overlaps could have occurred by chance (*p*-value). A very low *p*-value implies that the degree of overlap is highly significant statistically and very likely is significant biologically. In our study the gene lists we are comparing are the interactomes of lithium sensitive enzymes on the one hand, and KEGG pathways or otherwise derived lists associated with neural disease on the other hand. For each KEGG term retrieved, a null distribution of uncorrected *p*-value is generated by the 1,000 null sets. This gives us a measure of the false discovery rate, since any overlap between the null sets and the KEGG pathways is purely accidentally. Then the fraction of null set uncorrected *p*-values smaller than or equal to the lithium-sensitive set uncorrected *p-*value would be the empirical *p*-value. For a detailed discussion of empirical *p*-value determination see Ge et al. (2017).

### Key Gene Prediction

We predict key genes by counting how many times a gene appears in the cross section of interactomes and pathways associated with a particular disease. Then the counts are normalized by number of pathways associated with each disease. In this way, we predict which genes might be robust in disease-related pathways. Then, the genes are scored by the sum of mean counts over all diseases. A higher ranking indicates a gene would be associated with an important factor in many diseases.

## Results

**Figure 1:**
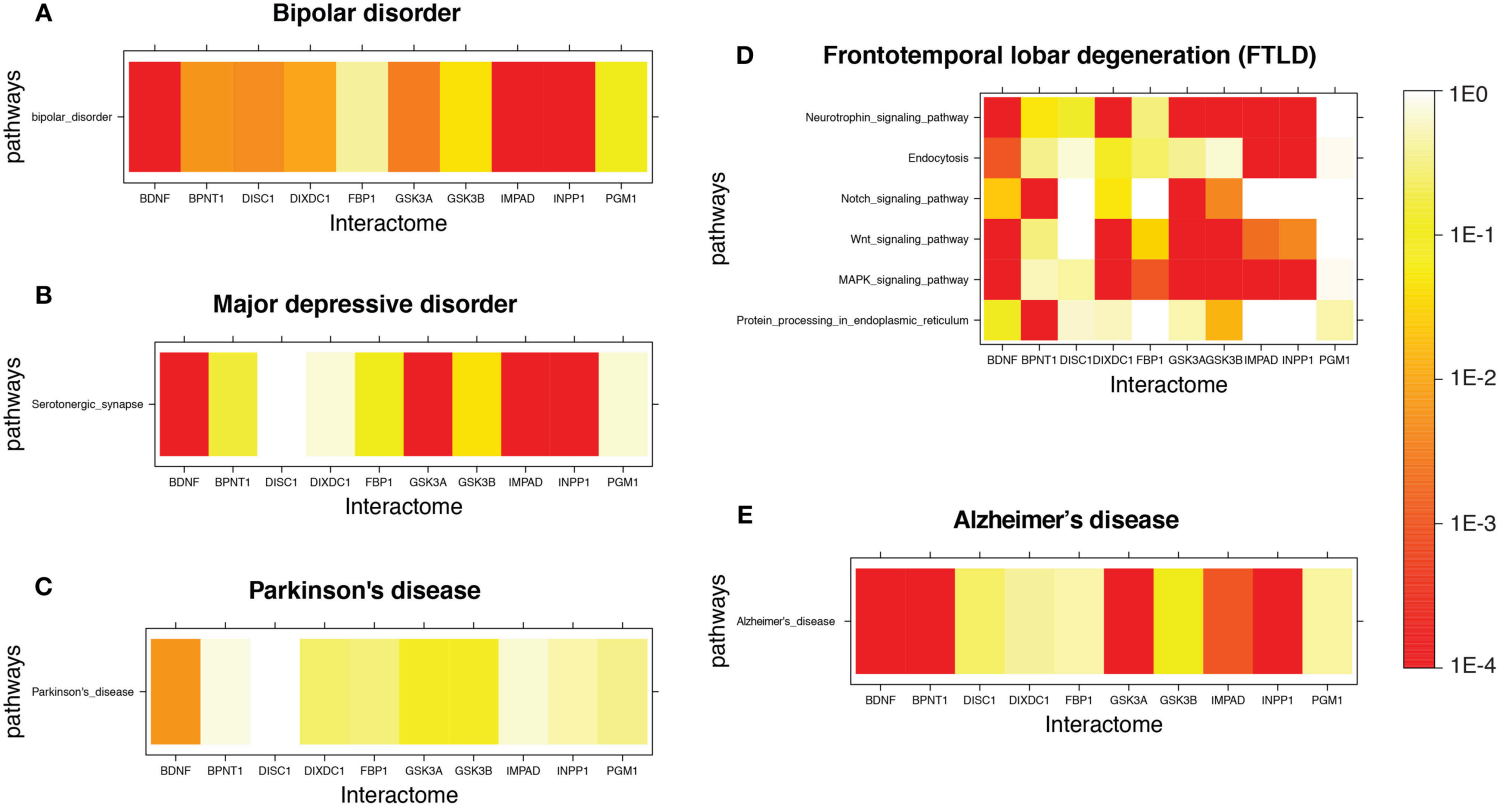
Heatmap for Lithium-sensitive enzyme interactome enrichment in disease-related pathways. The empirical enrichment *p*-value was calculated for each set of disease-associated genes. **(A,B)** are disorders where lithium treatment has proved to be effective and both show high enrichment. **(C,D)** are diseases where the effect of lithium treatment is unknown. **(C)** shows relatively low enrichment while **(D)** shows high enrichment. **(E)** is a disease where there is suggestive but not conclusive evidence for possible effectiveness of lithium therapy and shows high enrichment.

Figure 1 shows a few examples of mutual lithium interactome enrichment with specific disease pathways, represented by heatmaps. Each area on the heatmap is a color-coded representation of the degree of mutual enrichment between the genes in the interactome of the indicated lithium sensitive enzyme and the genes in the indicated pathway. The darker the shade, the more significant the mutual enrichment of the interactome-pathway combination is. Figures 1A,B shows two diseases where lithium treatments have been effective, and both show very strong enrichment. Figures 1C,D shows two diseases where the effect of lithium treatment has not been explored. Parkinson's disease, Figure 1C, shows low enrichment while FTLD, Figure 1D), shows high enrichment. We infer that FTLD is a more likely disease target for lithium treatment than Parkinson's Disease. Figure 1E) shows the heatmap for AD, for which evidence is strongly suggestive but not absolutely conclusive. A spreadsheet providing *p*-values for the mutual enrichment of the lithium sensitive interactomes and the relevant pathways for all 112 diseases studied are provided in Supplementary Material. We note that a few of the diseases.

For example, cataracts, are not always classified as neurodegenerative. However, in the KEGG disease database, sensory system diseases are a subset of the category “nervous system diseases.” Therefore, we did not exclude eye diseases such as cataracts, familial exudative vitreoretinopathy, and ring dermoid of cornea. The listing should be considered “diseases of the nervous system.”

For each of the 112 diseases we wished to compute a single number representing the relative likely sensitivity of the disease to lithium, in order to contribute to prioritizing which diseases are most likely to benefit from clinical trials with lithium. There is a significant literature on combining *p*-values (Loughin, 2004) with choices among methods depending on the detailed structure of the data. We adopt a relatively simple approach, which is to compute the geometric mean of the individual *p*-values for each pathway-interactome mutual enrichment value.

(1) $$\begin{array}{l} {\text{p}}_{\text{mean}}={\left({\text{p}}_{1}\times {\text{p}}_{2}\times {\text{p}}_{3}\times \ldots \ldots {\text{p}}_{\text{n}}\right)}^{1/\text{n}} \end{array}$$

The method of averaging in Equation (1) ensures that both strong and weak enrichments contribute significant weight to the mean. Note that all of the *p*-values that go into Equation (1) are corrected for false discovery rate by random resampling. Thus, no further false discovery rate correction is necessary for computing p_mean_. Note also that our method is bounded at the low end of *p*-values by the number of null samples it is reasonable to compute, given compute time constraints. For 1,000 null sets as used in this paper, the computed *p*-value will be zero when none of the thousand null sets shows the degree of enrichment of the test sets. For purposes of computing the p_mean_ in equation (1) we substitute 10^−4^ for zero for each of these cases.

Table 2 shows the top 34 ranked diseases out of the 112. Note that two diseases for which lithium is known to be effective therapy, bipolar disorder, and major depression disorder, rank high, 9 and 29, respectively. Other notable diseases shown in Table 2 include Alzheimers (20 out of 112), for which there is epidemiological evidence (Nunes et al., 2007) above, FTLD (30 out of 112) for which there is evidence via case history (Monji et al., 2014), and schizophrenia (22 out of 112) for which there is some evidence of efficacy as an adjunct to antipsychotics (Leucht et al., 2007). Scores for all 112 diseases are provided in Supplementary Material. The table also displays a “lithium sensitivity index,” which is 1/p_mean_.

**Table 2:** Top 34 neuron-related disease by lithium sensitivity.

**Disease**	**Sensitivity index (1/p_**mean**_)**	**Mean *p*-value**
1. Dravet syndrome	1718.943899	0.0006
2. HTLV1-Associated Myelopathy (HAM)	626.8531541	0.0016
3. Congenital pain insensitivity with anhidrosis	466.9837537	0.0021
4. Hemorrhagic destruction of the brain, subependymal calcification, and cataracts	418.143026	0.0024
5. Rasmussen encephalitis	293.1481892	0.0034
6. Lattice corneal dystrophies (LCD)	263.6451883	0.0038
7. Subependymal giant cell astrocytoma	246.0470815	0.0041
8. Bipolar Disorder	239.2876	0.0042
9. Familial episodic pain syndrome (FEPS)	231.5937968	0.0043
10. Familial exudative vitreoretinopathy (FEVR)	205.3474156	0.0049
11. Focal dermal hypoplasia	205.3474156	0.0049
12. Choroid plexus papilloma	198.0197413	0.0051
13. Juvenile-onset dystonia	183.6715435	0.0054
14. Prion diseases	175.0031999	0.0057
15. Axenfeld-Rieger syndrome (ARS)	169.1174332	0.0059
16. Congenital stromal corneal dystrophy (CSCD)	169.1174332	0.0059
17. Ring dermoid of cornea	169.1174332	0.0059
18. Stapes ankylosis with broad thumb and toes	169.1174332	0.0059
19. Benign familial neonatal and infantile epilepsies	153.4488999	0.0065
20. Alzheimer's disease	148.6362283	0.0067
21. Neurosis	132.0111986	0.0076
22. Schizophrenia	132.0111986	0.0076
23. Pituitary adenomas	123.9488444	0.0081
24. Febrile seizures	108.3195689	0.0093
25. Episodic ataxias	104.5457633	0.0095
26. Familial or sporadic hemiplegic migraine	104.5457633	0.0095
27. Cerebral amyloid angiopathy (CAA)	89.36992044	0.0112
28. Major depressive disorder	89.36992044	0.0112
29. Epileptic encephalopathy with continuous spike-waves during slow-wave sleep	87.85098473	0.0114
30. Frontotemporal lobar degeneration (FTLD)	75.82609324	0.0132
31. Cerebral palsy	72.92882566	0.0137
32. Generalized epilepsy and paroxysmal dyskinesia (GEPD)	69.3705138	0.0144
33. Amyotrophic lateral sclerosis (ALS)	67.25422275	0.0149
34. Fleck corneal dystrophy (FCD)	63.08690002	0.0158

**Figure 2:**
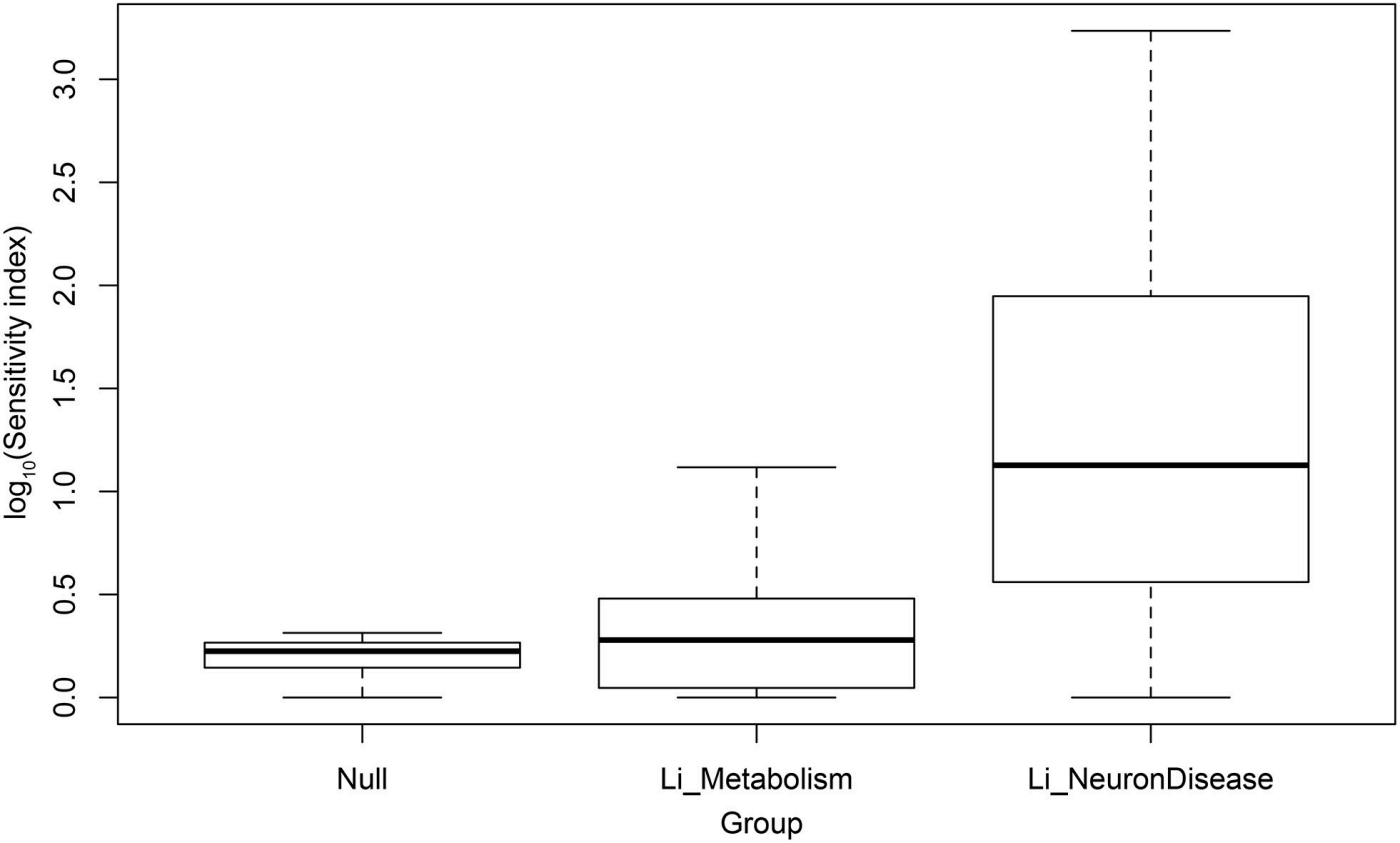
Log_10_ of sensitivity index of lithium-sensitive interactome for null sets, metabolic pathways, and pathways associated with disease of the nervous system.

As a control on our methods, we compared the statistical distribution of scores for neural disease with corresponding scores for metabolic pathways (also from KEGG), and with random gene sets (null sets). This comparison is shown in box plots in Figure 2. The vertical axis is the logarithm of the lithium sensitivity index. As expected the scores for the null sets are quite low, collapsing into a range between 1 and 2.05. The scores for the metabolic pathways are also low, reflecting fact that lithium has not been found to be major modulator of metabolism. Just two metabolic pathways account for the height of the upward extension of the metabolic box plot, carbohydrate metabolism and nucleotide metabolism. On the other hand, the scores for neural diseases are quite high. These scores, together with large numbers of cell, animal, and epidemiological studies suggesting lithium may play a role in ameliorating this class of disease, suggest moving forward into clinical trials for selected affective and neurodegenerative disorders. Even in studies in which lithium is not the primary variable, environmental lithium should be measured and correlated with outcomes or used as an experimental variable, because of the possibility that lithium and another drug may be synergistic. For example, lithium and rapamycin stimulate autophagy by independent pathways, leading to a suggestion that they might be a promising combination therapy for Huntington's disease (Sarkar et al., 2007).

In addition to pathways we examined our results to identify specific genes within the lithium sensitive interactomes that may be important in modulating lithium effect on disease. Table 3 indicates the genes that occur with the greatest frequency at the intersection of the lithium-sensitive interactomes and pathways associated with selected neural diseases. The complete tally for all 112 diseases considered in this study is provided in Supplementary Material. We suggest that genes that appear prominently at the intersection of lithium sensitivity and neural disease pathways, and their promoter regions, should receive attention as possible sites of important mutations affecting lithium response to neural disease, and possibly as targets for drugs to augment lithium in multidrug therapy. This is in addition to the 10 lithium-sensitive genes that were used as a starting point for this study, based on their previous mentions in the literature.

**Table 3:** Gene counts normalized by pathway number for genes appearing at intersection of interactomes and pathways.

	**Schizophrenia**	**Bipolar**	**AD**	**ALS**	**FTLD**	**Prion**	**MDD**	**Sum**
MAPK3	0	0	**4**	0	**1.33**	**4**	**4**	13.33
APP	0	0	**6**	0	0	0	**6**	12
TP53	0	0	0	**5**	**2.5**	0	0	7.5
RAC1	0	0	0	**4**	**2**	0	0	6
PSEN1	0	0	**4**	0	**2**	0	0	6
PLCB3	0	0	**3**	0	0	0	**3**	6
PLCB2	0	0	**3**	0	0	0	**3**	6
PLCB1	0	0	**3**	0	0	0	**3**	6
PPP3CC	0	0	**3**	**3**	0	0	0	6
PRKACB	0	0	0	0	0	**3**	**3**	6
ITPR1	0	0	**3**	0	0	0	**3**	6
PPP3CA	0	0	**3**	**3**	0	0	0	6
PRKACG	0	0	0	0	0	**3**	**3**	6
NOS1	0	0	**3**	**3**	0	0	0	6
PLCB4	0	0	**3**	0	0	0	**3**	6
PRKACA	0	0	0	0	0	**3**	**3**	6
CYCS	0	0	**3**	**3**	0	0	0	6
HTR2A	**1**	**2**	0	0	0	0	2	5
NOTCH1	0	0	0	0	0	**5**	0	5
GAPDH	0	0	**5**	0	0	0	0	5
MAP2K1	0	0	0	0	**0.67**	**2**	**2**	4.67
BAX	0	0	0	**2**	**0.67**	**2**	0	4.67
GRM1	**1.5**	**3**	0	0	0	0	0	4.5
TNF	0	0	**2**	**2**	0	0	0	4
GNAQ	0	0	**2**	0	0	0	**2**	4
GNG2	0	**2**	0	0	0	0	**2**	4
ITPR3	0	0	**2**	0	0	0	**2**	4
CDK5	0	0	**4**	0	0	0	0	4
FYN	0	0	0	0	0	**4**	0	4
IL1B	0	0	**2**	0	0	**2**	0	4
ITPR2	0	0	**2**	0	0	0	**2**	4
PRKCA	0	0	0	0	0	0	**4**	4
PPP3CB	0	0	**2**	**2**	0	0	0	4

*Table truncated for ease of display. Full table in Supplementary Material. We infer that these genes are interesting targets to augment lithium for potential multidrug therapy for the diseases indicated. All of the non-zero values are put in bold in order that they stand out from the “0” values*.

The genes in Table 3 are ranked by total number of appearance across the diseases. The high rank indicates that a gene might be (1) found associated with multiple diseases or (2) associated with multiple interactomes for a particular disease-associated pathway. For the former case, the gene might indicate similar mechanisms for the multiple diseases. For the latter case, the gene would be a promising target in treatment of that particular disease.

For example, MAPK3 is a shared gene by Alzheimer's Disease (AD), Prion, Major Depressive Disorders (MDD), and Frontotemporal lobar degeneration (FTLD), indicating that these diseases might have some shared mechanism. MAPK3 appeared in 4 interactome-pathway cross-sections in AD, Prion and MDD, and on average associated with 1.33 interactome-pathway cross-section in FTLD. MAPK3 is an essential component of the MAP signal transduction pathway that carries signals from cell surface to the nucleus. In analysis of normal as compared to AD brain tissue, MAPK3 is one of a small number of genes found to have alternative promoter usage and splicing (Twine et al., 2011).

Another prominent gene in Table 3 is APP (amyloid precursor protein), which gives a strong signal in both AD and MDD (Major Depressive Disorder). Published studies implicate specific mutations in APP in incidence of AD (Julia and Goate, 2017), and implicate amyloid beta, the cleavage product of APP, in incidence of MDD (Pomara and Bruno, 2016). Lithium has well-established efficacy in the treatment of MDD (Bschor, 2014), and regulates the production of amyloid beta (Su et al., 2004). Taken together these findings suggest that influence of lithium on APP may be a common mode of action of lithium effect on both AD and major depressive disorder.

## Summary and Discussion

We have conducted a pathway and network analysis of the role of lithium in 122 neurodegenerative and affective disorders. We have found that for the large majority of such disorders, there is high mutual enrichment between the interactomes of lithium-sensitive enzymes and the pathways associated with those diseases, indicating that lithium is very likely to affect the course of the disease. We have also identified specific genes that exist frequently at the intersection of lithium-sensitive interactomes and neural disease pathways, suggesting these genes as possible targets for more specific drugs than lithium.

We hope that the results described in this paper and more detailed Supplementary Material will contribute to prioritizing and designing clinical trials of lithium for neural disease. To provide context for such prioritization and design, it is essential to take into account the ways in which lithium is unique, both as a pharmaceutical and as an ion that is ubiquitous in the environment, and therefore ubiquitous in the water and food we ingest (Jakobsson et al., 2017):

1. Unlike other ions, lithium is not regulated by selective membrane transport processes. Therefore, lithium concentration in both extracellular and intracellular compartments, rather than being roughly constant, is roughly proportional to lithium ingestion.
2. Unlike other pharmaceuticals, lithium is wildly non-selective in its biochemical effects. The major underlying mechanism for the lack of selectivity is lithium's general propensity to inhibit the many enzymes that have magnesium as a cofactor.
3. Unlike other pharmaceuticals, lithium is an essential nutrient. The question with lithium is not whether it should be ingested or not, but rather how much. Extreme lithium deprivation results in failure to thrive, while too much lithium is toxic.

In the light of all these factors, we suggest that the correct question to ask with respect to lithium and a particular disease is not, “Should lithium be administered for this particular disease?” but rather, “What is the optimum blood level of lithium for this individual, given his or her disease history, status, and genetic propensities?” Unlike other pharmaceuticals that are far more specific and inhibit or activate one or a small number of genes, the model for lithium action is that it alters the balance between a large number of interacting processes and pathways. Thus, a dose-response curve for lithium is likely to be highly non-linear and not always monotonic.

There are just a few well-established markers for optimum concentrations. For a patient with a reliable diagnosis of bipolar disorder a common target for optimality would be blood concentration of 0.8–1 mM. Significantly higher concentrations will result in acute toxicity, while significantly lower will result in loss of effectiveness. Epidemiological studies on bipolar patients who are, and are not, on lithium therapy suggest that this level also protects against AD. However, this level has some side effects when sustained for years or decades, namely an increased risk of kidney damage and lowered thyroid activity. Thus, for other conditions one would like to find lower effective concentrations; indeed, one would like to do that for bipolar disorder as well, perhaps by combining lithium with other mood stabilizers that act in a synergistic fashion, enabling the lithium dose to be reduced.

At the other end of the dosage scale, epidemiological evidence is compelling that geographical variations in concentration of lithium in the drinking water are correlated with incidence of Alzheimer's; the lower the lithium the higher the incidence of mania. It thus seems that for Alzheimer's, an optimum level of blood lithium would be higher than the naturally occurring range, but perhaps lower than the therapeutic dose for bipolar disorder in order to minimize possible side effects of the bipolar therapeutic dose.

Another important marker is provided by a study showing that over a 4-year period a lithium level of 0.25–0.4 mM of lithium (1/4–1/2 of the bipolar therapeutic dose) did not incur any renal damage (Aprahamian et al., 2014). This study suggests that clinical studies exploring low to medium-dose lithium could be undertaken with relatively minimal concerns for side effects.

One of the authors (EJ) is an elderly person (79) and has found the evidence cited above sufficiently compelling that he self-administers lithium calibrated to a blood level of 0.3–0.4 mM, in order to reduce the pace of age-related neurodegeneration and also as a possible protection against cancer. His outcome, however important it may be to him personally, has no statistical significance. We need a clinical study involving many subjects addressing the same question.

In general, it seems clear that whatever other studies are undertaken with respect to affective and neurodegenerative disorders, lithium blood levels should be monitored for all patients, since even geographical variations may have significant effects. The cost of adding lithium level to the routine blood tests is minimal, especially compared to the potential benefits. Beyond that, multiple studies should be undertaken in which low- to moderate-level lithium supplements are administered, since these are likely to be safe (although of course side effects should be monitored and more extensive safety tests conducted).

Perhaps our results, especially as scored in Table 2 and combined with other considerations, might help to focus on which neurodegenerative diseases might be most useful to consider for lithium therapy. Other considerations might be: (1) whether the disease impacts a large number of people, so that alleviating the condition would relieve much suffering, (2) the age at which the condition strikes, considering that the impact on individual, family, and others may be more if the disease strikes at a younger age, (3) the mortality rate, and (4) how rapidly the disease progresses, since the more rapidly progressing the disease the more rapidly meaningful statistics may be gathered from an intervention trial.

Many conditions that score highly in Table 2 might be usefully considered. One condition that looms large to us, because of the loss of a person close to one of us at the age of 46, is FTLD. The mean *p*-value for FTLD pathway mutual enrichment with lithium-sensitive interactomes is.0132, which is highly significant. While not as common as Alzheimer's, FTLD is not rare. Estimated lifetime risk is 1/742; many millions of people each year die of FTLD (Coyle-Gilchrist et al., 2016). A ratio of official incidence to mortality is 0.97; FTLD is generally accepted to be 100% lethal. Life expectancy after diagnosis depends on the variant, but ranges from 3 to 9 years, so progression is much more rapid than Alzheimer's, permitting meaningful statistical analysis of any clinical trial in a relatively short time. Age of onset is most typically middle- to late middle-age when the individual is still employed and a crucial part of nuclear and extended family, in contrast to typically later onset of Alzheimer's. We have noted earlier in this paper that the initial symptoms of FTLD are sufficiently similar to mania (which is treated successfully with lithium) to sometimes lead to confusing diagnoses, which may indicate a common underlying biochemistry.

We will be happy to collaborate on further specific pathway or network analysis relevant to any of the neural diseases for which lithium may be a promising component of therapy.

## Author Contributions

The work was planned jointly in conversations between EJ and WG. WG did the computations and prepared the figures and tables. WG wrote the first draft of the Methods and Results sections. EJ wrote the first draft of the Introduction and Conclusions sections. Both authors shared in the final refinement of the manuscript.

### Conflict of Interest Statement

The authors declare that the research was conducted in the absence of any commercial or financial relationships that could be construed as a potential conflict of interest. The reviewer VDJRDP and handling editor declared their shared affiliation at the time of the review.

## Footnotes

^1^https://clinicaltrials.gov/ct2/show/NCT02862210
